# Constant versus seasonal photoperiod and temperature yield divergent life-history responses to climate warming

**DOI:** 10.1098/rspb.2026.1212

**Published:** 2026-10-28

**Authors:** Frank Johansson, Robby Stoks, Guillaume Wos, Szymon Sniegula

**Affiliations:** ^1^ Department of Ecology and Genetics, Ecology and Genetics, Uppsala, Sweden; ^2^ Department of Biology, KU Leuven, Leuven, Belgium; ^3^ Institute of Nature Conservation Polish Academy of Sciences, Krakow, Poland

**Keywords:** developmental timing, ectotherms, experimental ecology, phenotypic plasticity, photoperiodism, seasonal cues, thermal variation

## Abstract

A key goal of predictive ecology is to understand how global warming affects ectotherm growth and development. Researchers often rear organisms under constant temperature and photoperiod regimes, raising the question of whether responses under these simplified conditions reflect those in realistic, seasonally varying environments. We examined key life-history traits of the damselfly *Lestes sponsa* across six combinations of constant and seasonal temperatures and photoperiod regimes, simulating present-day and future climates with a projected 4°C warming. Trait responses differed qualitatively and quantitatively among treatments. Mass and development responses to warming shifted direction depending on regime. For example, the response in mass to warming was reversed between constant (decrease) and seasonal varying (increase) conditions. In addition, developmental synchrony was stronger under seasonal photoperiods, and the temperature–size rule held only under constant conditions. Our results highlight that incorporating seasonal variation is crucial for developing a realistic framework for predicting species' responses to climate change.

## 1. Introduction

One key goal in predictive ecology is to understand how global temperature change affects organismal growth and development, particularly in ectotherms, which rely on external temperatures to regulate their body temperature [1]. Growth-rate determines size at maturation, influencing mating success and reproductive output [2,3], whereas development time affects the timing and duration of reproduction as well as synchrony with seasonal resources [4–6]. Photoperiod is another environmental factor affecting growth and development. Unlike temperature, photoperiod remains unchanged by climate warming at a given latitude [7,8], although climate-driven shifts in phenology can expose organisms to novel photoperiod regimes [9–11]. Together, temperature and photoperiod regulate the timing and synchrony of emergence and maturation in ectotherms [12–14], which is crucial for time-constrained species that must complete development under favourable conditions. Consequently, experiments relying on constant conditions or examining temperature and photoperiod in isolation may bias estimates of growth, development and synchronization. Although many studies have investigated the effects of temperature or photoperiod separately [15–21], to our knowledge, no studies have examined their combined effects under realistic seasonal conditions simulating contemporary environments and projected global warming scenarios.

A well-established pattern in ectotherms is the temperature–size rule, which states that individuals developing at higher temperatures often grow faster but mature at a smaller size [22]. This pattern arises because warming accelerates development more strongly than growth. However, the expression of the temperature–size rule may depend on photoperiod, which affects developmental timing and can alter growth trajectories [23]. These responses reflect life-history trade-offs arising from limited energy availability, whereby increased investment in one trait often reduces investment in another [24]. For example, higher temperatures or photoperiod-imposed time constraints typically lead to a lower mass at maturation [24]. Understanding how these trade-offs differ between constant and seasonally varying environmental conditions is therefore important for predicting organismal responses to climate change.

The effects of temperature on life-history traits have been studied extensively in laboratory experiments [9,10,25–30]. However, it remains unclear how well the results of such experiments predict growth and development under natural conditions. Many studies have used constant temperatures and photoperiods [11,15,29], despite natural environments being characterized by seasonal variation [31,32]. In temperate regions, the photoperiod increases early in the growing season, peaks around midsummer and then declines. In many ectotherms, photoperiod provides a reliable cue of seasonal progression and strongly influences growth and development in insects, amphibians and reptiles [7,8,26,33]. A decreasing photoperiod signals a reduced time available for development and can induce accelerated growth, shorter development or initiation of winter diapause, depending on species-specific seasonal strategies [34]. These responses differ from those expressed earlier in the season when photoperiod increases [7]. Although some studies have employed naturally varying temperature and photoperiod [35], direct comparisons with constant temperature and/or photoperiod treatments remain rare.

Damselfly larvae (*Lestes sponsa*) are excellent model ectotherms for studying whether life-history responses observed under constant temperature and photoperiod accurately represent those occurring under naturally fluctuating seasonal conditions. Their extensive use in past climate change studies [9,15,27,36,37] further highlights their value as model organisms. The aquatic larval stage lasts from weeks to months, followed by a terrestrial stage for mating and egg-laying. Adult body size is fixed at emergence, and although adults may subsequently gain mass, individuals emerging at low mass generally remain lighter as mature adults [38]. Because adult size is a key fitness component [39,40], larval growth strongly influences adult success. Emergence timing also affects fitness, as early emerging individuals with longer lifespans achieve higher reproductive success [41].

Our main aim was to assess whether life-history responses under constant temperature and photoperiod conditions reflect those observed under naturally varying seasonal conditions. To address this question, we compared development time, growth-rate, mass and synchrony of emergence across six treatments simulating ambient and warming temperature regimes under both constant and seasonally changing temperatures and photoperiods (table 1). Treatments with seasonally changing temperature and photoperiod mimicked post-winter conditions in temperate regions.

**Table 1.** Overview of the six treatments with different combinations of temperature (temp) and photoperiod (photop), including treatment descriptions and predicted rankings of growth-rate (highest to lowest) and development time (shortest to longest). Note that we ended the experiment when all larvae reached the F-0 instar. Since the last larva in the seasonal ambient temp + seasonal photop treatment reached the F-0 instar earlier than in the seasonal ambient temp + constant photop treatment, the seasonal ambient temp + seasonal photop treatment experienced a lower average temperature.

treatment and abbreviations used in the text	abbreviations used in figures 1–2	temperature profile	photoperiod profile	notes	predicted ranking of growth-rate and development time from highest to lowest and shortest to longest (1–6)
constant ambient temp + constant photop	CAT+CP	constant 20.3°C	constant L–D 14–10 h	moderate temperature, no seasonal photoperiod cue	4. Similar temperature to in treatment constant ambient temp + seasonal photop, but no photoperiod signal, so lower growth-rate and longer development compared with the constant ambient temp + seasonal photop.
constant warmer temp + constant photop	CWT+CP	constant 24.5°C (warmer temperature)	constant L–D 14–10 h	warmer temperature, no seasonal photoperiod cue	2. High constant temperature near thermal optimum, no photoperiod cue, but favourable high temperature alone should cause the second fastest growth-rate and the second shortest development.
seasonal ambient temp + seasonal photop	SAT+SP	seasonal increasing 8.5–21.9°C (ambient temperature)	seasonal increasing from L–D 14.03–9.57 h to 18.51–5.09 h	natural seasonal temperatures and photoperiod cues	5. Realistic spring simulation. Lower temperature than in the constant ambient temp + constant photop treatment with an average of 17.3°C, but photoperiod increases, which probably compensates somewhat by signalling urgency to develop.
seasonal warmer temp + seasonal photop	SWT+SP	seasonal increasing 12.8–24.3°C (seasonal warming temperature)	seasonal increasing from L–D 14.03–09.57 h to 18.23–5.37 h	seasonal warming scenario, with natural seasonal temperatures and photoperiod cues	1. Moderately high temperature, average over all weeks is 19.9°C, and a photoperiod signal of seasonal progression; should expect the fastest growth-rate and the shortest development.
constant ambient temp + seasonal photop	CAT+SP	constant 20.5°C	seasonal increasing from L–D 14.03–09.57 h to 18.51–5.09 h	moderate temperature, seasonal photoperiod cue	3. Moderate temperature, but increasing photoperiod, should expect high growth and short development despite not being thermally optimal.
seasonal ambient temp + constant photop	SAT+CP	seasonal increasing 8.5–22.7°C (ambient temperature)	constant L–D 14–10 h	seasonal temperature, no seasonal photoperiod cue	6. Cool treatment (especially early in the experiment) with an average of 17.9°C and no photoperiod cue, so the lowest growth-rate and the longest development expected.

We predicted that growth-rate and development time would vary among treatments as a function of both temperature and photoperiod. Because ectotherms in temperate regions commonly experience seasonal changes in temperatures and photoperiods, which we simulated in our experiment, and because temperature- and photoperiod-dependent responses in growth and development are generally consistent across ectotherm taxa, we expected that our results would be broadly applicable to temperate arthropods, amphibians and reptiles. Based on the thermal and photoperiod conditions, we developed a set of specific predictions (table 1). We expected the fastest growth and shortest development time under seasonal warming temperatures combined with a seasonal photoperiod, where favourable thermal conditions coincide with a strong seasonal cue (seasonal warmer temp + seasonal photop; table 1). We expected the second highest growth-rate and second shortest development time under constant warmer temperature with a constant photoperiod, driven primarily by optimal thermal conditions (constant warmer temp + constant photop; table 1). We predicted intermediate responses under a constant ambient temperature combined with a seasonal photoperiod, because photoperiodic cues may partially compensate for suboptimal temperatures (constant ambient temp + seasonal photop; table 1), followed by constant ambient temperature and a constant photoperiod, where such cues are absent (constant ambient temp + constant photop; table 1). We predicted lower growth-rates and longer development times under seasonal ambient temperatures combined with a seasonal photoperiod, reflecting overall cooler conditions (seasonal ambient temp + seasonal photop; table 1). We expected the slowest growth and longest development under seasonal ambient temperatures combined with a constant photoperiod (seasonal ambient temp + constant photop; table 1). Finally, we predicted greater emergence synchrony under seasonally increasing photoperiod as it provides a reliable cue of seasonal time constraints.

## 2. Material and methods

### (a) Study species

To study growth and developmental responses to temperature and photoperiod, we raised larvae of the damselfly *Lestes sponsa* under different temperature and photoperiod scenarios. *L. sponsa* is widespread across Europe [42]. It exhibits a univoltine life cycle with distinct aquatic and terrestrial phases, comprising overwintering egg, aquatic larval and a short-lived terrestrial adult stage [43]. Egg hatching takes place in spring, and larval development proceeds over approximately 1.5–3.0 months, depending on environmental factors such as temperature and photoperiod [14,20,44,45]. Owing to seasonality, larvae experience directional selection for rapid growth and development rates [46]. Traits expressed at emergence, such as body size and mass, are known to influence key fitness-related parameters in adults [47]. There is no information on the thermal optimum for growth in *L. sponsa*, but a previous study indicated that growth at 23°C was more favourable than at 21°C [37], and conspecific *Lestes disjunctus* has a thermal optimum close to 24.8°C [48].

### (b) Adult field collection, egg-laying and larval rearing

Mated adult females, 23 individuals in total, were collected from a pond in north-western Poland (53°39′27.6″ N 16°16′26.6″ E) on 6 August 2024. Females were individually enclosed in jars with moisturized filter paper for egg-laying. Jars with females were placed in a room at 20°C and the natural photoperiod for another four days during which egg-laying happened. In total, 20 females laid egg clutches that were used in the experiment. The egg clutches were then transported by car to the Institute of Nature Conservation PAS in Krakow, Poland, where the experiment was run. Upon arrival (12 August 2024), each egg clutch was divided into six equal parts, with one part from each clutch placed in a separate plastic container, so that each container held one-sixth of all clutches. The six containers, which later constituted the six treatments (described in detail in §2c), were placed in an incubator (Pol-Eko, ST 700) set to 19.9°C (water temperature) and a photoperiod of L–D 16.34–07.26 h (including civil twilight), mimicking the ambient conditions at the sampling site (supplementary material, table S1). Following an average of 32 days under progressing summer and autumn temperatures and photoperiod regimes, the containers with clutches were thereafter transferred to overwintering within the same incubator. The overwintering treatment began on 13 September 2024 and was maintained at a constant temperature of 7.0°C (water temperature reflecting ambient air temperature) in complete darkness. Under these conditions, the clutches were overwintered for 22 weeks. Although these conditions do not fully replicate natural winter conditions, they represent a standardized and commonly used approach to maintain diapause and ensure synchronous overwintering of eggs across clutches [45], thereby avoiding variation unrelated to the experimental treatments. After such wintering conditions and duration, eggs may hatch either towards the end of the wintering period or with the onset of spring temperature and photoperiod [44,49]. Thereafter, we simulated spring conditions by setting the temperature and photoperiod to match the timing when water temperatures exceeded 8°C, which corresponds to 28 March at the population of origin (supplementary material, table S2). By this time, many individuals were in the hatchling stage (the first larval instar). These hatchlings were used in the experiment. Larvae were reared individually in plastic cups (7 cm in diameter and 4 cm in height) and fed daily with laboratory-cultured brine shrimps *Artemia* sp. twice a day during weekdays and once a day during weekends. If an experimental larva died, it was replaced by a backup individual of the same age and size (= instar). Backup larvae were reared in the same incubator as the experimental larvae; hence, they experienced the same temperature and photoperiod conditions. Replacements were made until the first experimental larvae reached the pre-final instar before emergence (F-1) to avoid bias between individually reared (experimental) and group-reared (backup) larvae. In our statistical analyses (see §2e), we ran two separate tests for each variable: one including backup larvae and one excluding them. The results of the two tests did not differ qualitatively (statistics not shown). Therefore, the results presented below are based on an extended dataset, including replaced larvae from the backup. Water temperature in each treatment was regulated by programming the corresponding air temperature in the incubator. Generally, water temperatures were *ca* 1°C lower than air temperatures; see table S3 in the supplementary material for the achieved mean, minimal and maximal water temperatures per treatment. Detailed descriptions of daily water temperatures per treatment are in table S2 in the supplementary material. The experiment ended when the last individual entered the final instar before emergence (F-0).

### (c) Larval treatment description

We established six temperature–photoperiod treatments, each starting with 50 individuals (table 1). Owing to the limited number of climate chambers, a full factorial design was not possible. The treatments were:

— Constant ambient temp + constant photop: Baseline treatment with a constant water temperature of 20°C and a photoperiod of L–D 14–10 h, commonly used in insect development studies [50–54].
— Constant warmer temp + constant photop: Constant 24°C and L–D 14–10 h, representing a 4°C warming scenario relative to the baseline.
— Seasonal ambient temp + seasonal photop: Natural spring conditions starting on 28 March, with temperature and photoperiod changing weekly.
— Seasonal warmer temp + seasonal photop: As above, but with a 4°C increase in weekly temperatures to simulate warming.
— Constant ambient temp + seasonal photop: Constant 20°C combined with a seasonally changing photoperiod.
— Seasonal ambient temp + constant photop: Seasonally changing temperature combined with a constant photoperiod of L–D 14–10 h.

Table 1 presents the predicted treatment rankings for growth-rate and development time. Seasonal temperature treatments were, on average, more than 2°C colder than the corresponding constant temperature treatments (table 1; supplementary material, tables S2 and S3).

### (d) Response variables

Five life-history traits were quantified: survival, development time, body mass, growth-rate and developmental synchrony (variation in timing of entry into the F-0). Development time was defined as the number of days from hatching until entry into the F-0. Wet body mass was measured at the onset of the F-0 using an electronic balance to the nearest 0.01 mg (Radwag^®^ AS.62. R2). Body mass at entering F-0 is highly correlated with size and mass at emergence in damselflies [55,56]. Given the established positive correlation between body size and mass in damselfly larvae [55], body mass at F-0 was used as a proxy for overall adult size. While we acknowledge that no proxy is perfect, mass has often been used as a proxy for size, including in meta-analyses on the temperature–size rule [57]. Growth-rate was calculated as the ratio of body mass at F-0 entry to the number of days of larval development. Growth-rate is generally considered a key fitness component in damselflies [58]. Developmental synchrony was assessed as the coefficient of variation (CV) in development time until the entry into the F-0 within each experimental treatment.

### (e) Statistical analyses

All statistical analyses were run in R [59]. Survival differences among treatments were analysed using a binomial generalized linear model (GLM) with a logit link function based on individual survival outcomes (backup larvae excluded). Normality of residuals was assessed visually using Q-Q plots. To examine the effects of treatment and sex on larval development time, mass and growth-rate, we fitted separate linear models with each trait as the response variable and treatment and sex as fixed effects. Interaction terms were initially included and removed from the final models if not significant. Developmental synchrony was estimated by calculating the CV for each treatment; lower CV values indicate more synchronous larval development. We used a global Fligner–Killeen test of homogeneity of variances to assess whether the variability in development time differed significantly across treatments. To examine the trade-offs among traits, we fitted linear models with mass as the response variable, development time as a covariate, treatment as a fixed factor and their interaction to test whether the relationship between development time and mass differed among treatments. Treatment-specific slopes were additionally visualized using regression lines, and model-based slope estimates for each treatment are reported in table S4 in the supplementary material.

## 3. Results

### (a) Survival and replacement

Before the first experimental larvae reached the F-1 instar, 7 (14%)–14 (28%) individuals per treatment died and were replaced with backup larvae. There was weak evidence for an association between treatment and survival, but the effect was not significant (binomial GLM: $\chi^{2}$ = 10.38, d.f. = 5, *p* = 0.065; backup larvae excluded). Survival probabilities tended to be higher in the constant ambient temp + constant photop, constant warmer temp + constant photop and constant ambient temp + seasonal photop treatments, and lower in the seasonal ambient temp + seasonal photop, seasonal ambient temp + constant photop and seasonal warmer temp + seasonal photop treatments, showing intermediate values (supplementary material, figure S1). By the time larvae reached the F-0 instar, i.e. at the end of the experiment, 40–50 individuals per treatment, including backups, had survived, and the subsequent analyses were based on these individuals.

### (b) Development time

Overall, a seasonal photoperiod appeared to promote shorter development when combined with warmer or constant temperature conditions. Additionally, higher temperatures tended to shorten larval development, while cooler, seasonally increasing temperatures prolonged it. More specifically, there was a significant effect of treatment on development time. The effect of sex was not statistically significant (*p* = 0.06), but females exhibited a tendency to have longer development times than males (figure 1a and table 2). Larvae in constant warmer temp + constant photop treatment (constant 24.5°C with a constant photoperiod) had the shortest development time, followed by constant ambient temp + seasonal photop, seasonal warmer temp + seasonal photop, constant ambient temp + constant photop, seasonal ambient temp + seasonal photop and those in seasonal ambient temp + constant photop treatment (seasonally increasing 8.5–22.7°C with a constant photoperiod) showing the longest development time (figure 1a). Thus, the constant warmer temp + constant photop treatment, which we predicted should have the second shortest development time, had the shortest development time, followed by the constant ambient temp + seasonal photop treatment, which we predicted should have the third shortest development time.

**Figure 1.**
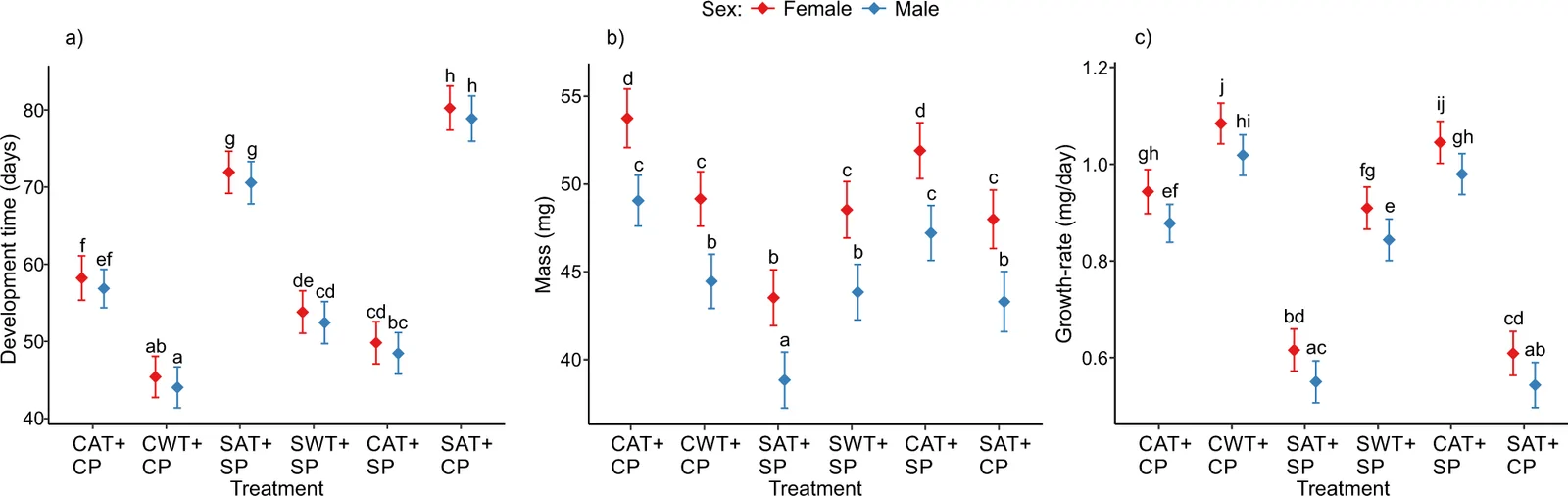
Estimated mean (a) development time, (b) mass and (c) growth-rate until entrance into F-0 in *L. sponsa*. Error bars show 95% CI. Abbreviations: CAT+CP—constant ambient temperature and constant photoperiod; CWT+CP—constant warmer temperature and constant photoperiod; SAT+SP—seasonal ambient temperature and seasonal photoperiod; SWT+SP—seasonal warmer temperature and seasonal photoperiod; CAT+SP—constant ambient temperature and seasonal photoperiod; SAT+CP—seasonal ambient temperature and constant photoperiod.

**Table 2.** Results from linear models for larval development time, mass and growth-rate until entrance into F-0, and from a model including an interaction between development time (covariate) and treatment for mass in *L. sponsa*. The interaction model tested whether the relationship between development time and mass differed among treatments. A significant interaction term (treatment × development time) indicates heterogeneity in slopes among treatments. The interaction treatment × sex was not significant and is not shown.

predictor	d.f.	*F*	*p*
**development time**			
treatment	5	226.13	<0.001***
sex	1	3.52	0.062
**mass**			
treatment	5	48.26	<0.001***
sex	1	122.50	<0.001***
**growth-rate**			
treatment	5	218.61	<0.001***
sex	1	32.28	<0.001***
**mass ∼ treatment × development time**			
treatment	5	9.15	<0.001***
development time	1	1.14	0.285
treatment × development time	5	9.45	<0.001***

Table shows degree of freedom (d.f.), *F*-values and *p*-values. Significance is indicated in bold *** *p* < 0.001.

### (c) Mass

In general, larval mass was influenced by both temperature and photoperiod, with a lower mass associated with seasonal temperature and photoperiod conditions. Notably, there was a significant effect of both treatment and sex on larval mass (figure 1b and table 2). Across all treatments, females exhibited a higher mass at emergence than males (figure 1b). The highest mass was observed in the constant ambient temp + constant photop treatment (constant 20.3°C, constant photoperiod) and in the constant ambient temp + seasonal photop treatment (constant 20.5°C, seasonal photoperiod), while the lowest mass occurred in the seasonal ambient temp + seasonal photop treatment (seasonally increasing 8.5–21.9°C, seasonal photoperiod). The remaining treatments resulted in intermediate mass values (figure 1b).

### (d) Growth-rate

Temperature generally had a stronger influence on growth-rate than photoperiod, with higher and more stable temperatures promoting faster growth, while photoperiod effects were more variable and dependent on the temperature regime. In detail, growth rate was significantly influenced by both treatment and sex, with females exhibiting higher growth-rates than males (figure 1c and table 2). The highest growth-rates were observed under constant temperature conditions (24 or 20°C) paired with either constant or seasonal photoperiods, respectively (constant warmer temp + constant photop and constant ambient temp + seasonal photop treatments; figure 1c). By contrast, the lowest growth-rates occurred under treatments with a cooler seasonal temperature regime, regardless of photoperiod (seasonal ambienttemp + seasonal photop and seasonal ambient temp + constant photop treatments; figure 1c). Intermediate growth-rates were found in treatments with constant 20°C or warmer seasonal temperatures combined with constant or seasonal photoperiods (constant ambient temp + constant photop and seasonal warmer temp + seasonal photop treatments, respectively; figure 1c).

### (e) Synchrony of larval development time

Synchrony of development time until entrance into F-0 was significantly affected by the treatments (Fligner–Killeen $\chi^{2}$ = 20.67, d.f. = 5, *p* < 0.001). The three treatments with seasonal photoperiod had higher synchrony than the treatment with a constant photoperiod (figure S2).

### (f) Trade-offs

The relationship between mass and development time differed among treatments. This was supported by a significant interaction between treatment and development time (table 2), indicating different slopes among the treatments (supplementary material, table S4). Visual inspection of the regression lines (figure 2) showed that some treatments exhibited positive relationships (constant warmer temp + constant photop and constant ambient temp + seasonal photop treatments), while others showed weak positive relationship (seasonal ambient temp + constant photop treatment), no relationship (constant ambient temp + constant photop treatment) or weak negative relationship (seasonal warmer temp + seasonal photop and seasonal ambient temp + seasonal photop treatments).

**Figure 2.**
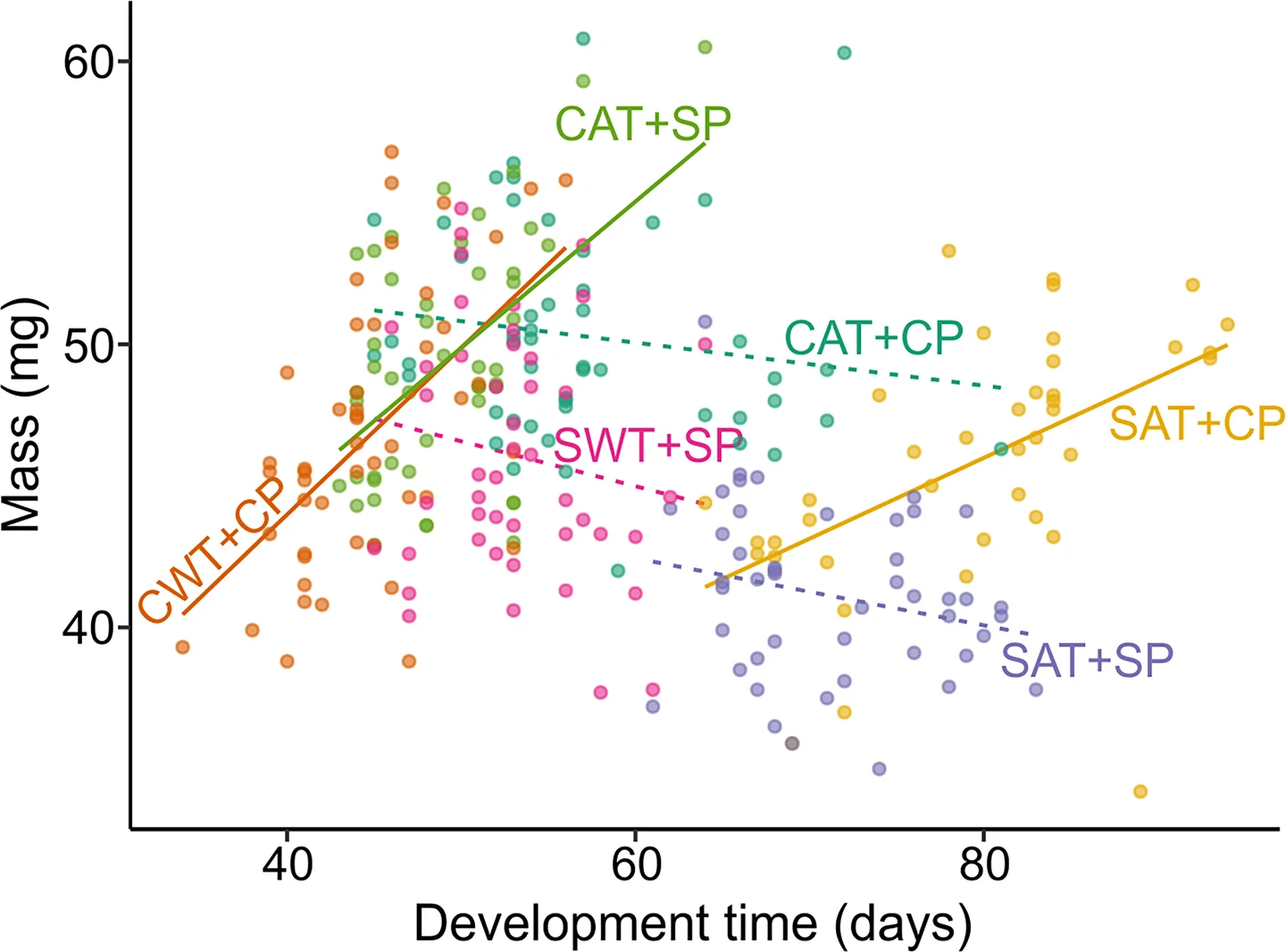
Relationship between mass and development time across treatments in *L. sponsa*. Points represent individual larvae, and lines show treatment-specific regression relationships. Solid lines indicate statistically significant relationships, whereas dashed lines indicate non-significant relationships (added for visual interpretation of statistically significant interaction terms). Abbreviations: CAT+CP—constant ambient temperature and constant photoperiod; CWT+CP—constant warmer temperature and constant photoperiod; SAT+SP—seasonal ambient temperature and seasonal photoperiod; SWT+SP—seasonal warmer temperature and seasonal photoperiod; CAT+SP—constant ambient temperature and seasonal photoperiod; SAT+CP—seasonal ambient temperature and constant photoperiod. See table S4 in the supplementary material for slope estimates.

## 4. Discussion

Understanding how temperature affects insect growth and development is essential for predicting population responses to environmental change. Many studies have examined this by rearing insects at different temperatures in controlled laboratory settings [10,60,61]; however, most studies used constant temperatures and photoperiods, which may be problematic because such conditions can elicit responses that deviate from those generated by more realistic environmental fluctuations. By applying multiple temperature and photoperiod regimes to a single species and population, our study investigated how the choice of experimental regime shapes key life-history responses. This raises an important question for future research: which experimental regime is most appropriate when aiming to predict the effects of global warming on ectotherm life history?

### (a) Choosing the right warming and photoperiod treatment

To assess the impact of climate warming, a common approach is to compare a constant ambient temperature with a constant warmer temperature under a constant photoperiod [10,62,63]. This corresponds to the comparison between our constant ambient temp + constant photop treatment and the constant warmer temp + constant photop treatment, which revealed a 21.8% decrease in development time, a 7% decrease in mass and a 17.3% increase in growth-rate at the warmer temperature, thereby supporting the temperature–size rule. By contrast, we also assessed the effect of climate warming under seasonal temperature and photoperiod regimes, i.e. comparing seasonal ambient temp + seasonal photop with seasonal warmer temp + seasonal photop, which revealed a 25.4% decrease in development time, an 11.9% increase in mass and a 50.1% increase in growth-rate for the global warming scenario. Hence, the responses in development time and growth-rate differed quantitatively between approaches, whereas mass differed both quantitatively and qualitatively, showing opposite responses to warming. In light of these differences, incorporating seasonal variations in both temperature and photoperiod may offer a more ecologically realistic framework for predicting responses to climate change in time-constrained ectotherms. Because we found both quantitative and qualitative differences in responses to warming depending on the experimental approach, we suggest that seasonal temperature and photoperiod regimes simulating natural seasonal progression should be used [64,65], particularly for organisms with fixed voltinism.

Taken together, our results reveal distinct and sometimes decoupled effects of temperature and photoperiod on larval development time, mass, growth-rate and synchrony. Responses in development time, mass and growth-rate were not always aligned; changes in development time did not consistently correspond to changes in mass. For example, the seasonal ambient temp + constant photop treatment (seasonal 8.5–22.7°C, constant photoperiod) had the longest development time but an intermediate mass, whereas the constant ambient temp + constant photop treatment (constant 20°C, constant photoperiod) produced the largest mass with an intermediate development time.

### (b) Development and growth-rate

We predicted that the seasonal warmer temp + seasonal photop treatment would result in the shortest development time and fastest growth-rate, followed by the constant warmer temp + constant photop treatment. Our results did not support this prediction for development time because constant warmer temp + constant photop had the shortest development time, probably owing to the higher temperature in this treatment compared with the seasonal warmer temp + seasonal photop treatment. In addition, constant ambient temp + seasonal photop (constant 20°C, seasonal photoperiod), which we predicted would have the third shortest development time and third fastest growth-rate, did not differ from the seasonal warmer temp + seasonal photop treatment in development time. Interestingly, despite constant warmer temp + constant photop being 4°C warmer than constant ambient temp + seasonal photop, their growth-rates did not differ significantly, suggesting that photoperiod strongly regulates growth. Alternatively, the thermal optima for development and growth may differ [66], or larvae may reach physiological limits, preventing further acceleration of growth. Photoperiod is known to signal seasonal progression and shorten development time in damselfly larvae [9,33,67,68], and both treatments that included a seasonal photoperiod, seasonal warmer temp + seasonal photop and constant ambient temp + seasonal photop, showed relatively short development times. Although temperature also influenced development time, this pattern suggests that photoperiod plays an important role in regulating this life-history trait.

We also predicted that the treatment seasonal ambient temp + constant photop (seasonal 8.5–22.7°C, constant photoperiod), which involved no warming, would result in the longest development time and the lowest growth-rates. Our results support this prediction, probably driven by the relatively low mean temperature (17.9°C) and the absence of a seasonal photoperiod signalling seasonal progression. These results are consistent with earlier studies showing longer development and slower growth at lower temperatures and under photoperiods that do not mimic late-season conditions [49,67]. Notably, the treatment seasonal ambient temp + seasonal photop (seasonally increasing 8.5–21.8°C, seasonal photoperiod) had a shorter development time than the seasonal ambient temp + constant photop treatment, despite having a slightly lower mean temperature (supplementary material, table S3). This again highlights the important role of photoperiod in regulating development. However, since growth-rate did not differ significantly between these two treatments, the influence of photoperiod may be limited in this comparison, potentially offset by the higher average temperature in seasonal ambient temp + constant photop treatment.

In summary, the results on development time and growth-rate suggest that high temperatures shortened development time and accelerated growth, even under constant photoperiod. A seasonal photoperiod promoted a shorter development time under seasonal temperature regimes compared with constant photoperiod. We did not include the constant warmer temp +** **seasonal photop treatment, which would have allowed a direct comparison with the constant warmer temp + constant photop treatment.

### (c) Temperature–size (mass) rule

The temperature–size rule proposes that ectotherms developing at lower temperatures tend to have longer development times and reach a larger adult size [22]. Interestingly, both treatments with the constant ambient temperature, constant ambient temp + constant photop and constant ambient temp + seasonal photop, led to the highest mass, but not to the longest development time. Instead, both treatments with the seasonal ambient temperature, seasonal ambient temp + seasonal photop (seasonal 8.5–21.9°C, seasonal photoperiod) and seasonal ambient temp + constant photop (seasonal 8.5–22.7°C, constant photoperiod) had the longest development time but low and intermediate mass, respectively. Thus, we found no overall support for the temperature–size rule, suggesting that the commonly reported patterns for temperate species may be biased, as most studies exploring the relationship between development and mass have relied on constant temperatures and photoperiods. Several factors could explain this discrepancy with respect to the temperature–size rule. First, in the two treatments with the seasonal ambient temperature (seasonal ambient temp + seasonal photop and seasonal ambient temp + constant photop), colder phases occurred during the early larval stages, which probablyslowed metabolism and growth. This temperature effect in the early stages of development could reduce overall growth efficiency and net biomass accumulation despite the longer growth duration. A second non-mutually exclusive explanation could be that the constant photoperiod does not represent a reliable cue of season, i.e. the treatment seasonal ambient temp + constant photop may represent a mismatched cue scenario. Here, temperatures change as in nature, but photoperiod does not reflect the seasonal progression. This may cause developmental asynchrony, stress or suboptimal growth patterns, leading to intermediate performance and lower mass.

### (d) Synchrony

In time-constrained ectotherms, the synchrony of hatching and maturation is fundamental because their timing must align with the optimal conditions for feeding, reproduction and dispersal. Photoperiod is a crucial and more predictable environmental cue than temperature that synchronizes hatching and maturation in insects [8]. We predicted that emergence synchrony would be higher under seasonally increasing photoperiod, as it provides a reliable cue of seasonal time constraints. In support of this, our results showed that synchronization of development was higher under seasonal photoperiod in all treatments, and this was independent of temperature treatment. Therefore, we recommend the use of a seasonal photoperiod when investigating the effects of climate change on the timing and coordination of life-history events in ectotherms because using a constant photoperiod may lead to asynchronous development and growth. This temporal misalignment could be especially important for species with fixed voltinism experiencing a time-constrained season.

### (e) Sex differences

As *L. sponsa* females are generally larger than males and emerge later [69], it is expected that females exhibit higher growth-rates and/or longer development times, while males develop more quickly in this species. In the context of rising temperatures, females are predicted to be more responsive to thermal changes, potentially showing greater shifts in both growth and development time [70]. We found support for higher growth-rate and greater mass in females and a trend towards longer development time in this sex, as found in several other *Lestes* species [37,67]. However, we did not find support for a relatively higher growth-rate in females than in males at the higher temperature, as there was no significant interaction term between sex and treatment (interaction: *F*₅,₂₆₆ = 1.16, *p* = 0.329). One possible explanation for this lack of sex-specific thermal plasticity is intralocus sexual conflict. Intralocus sexual conflict arises when the same genes are subject to opposing selection pressures in males and females, constraining each sex's ability to independently optimize trait expression [71]. This constraint may be particularly strong in seasonal species or populations, where strict seasonality imposes strong selection for timely development in both sexes. In such environments, selection for rapid and synchronous development may override or mask sex-specific selection pressures on growth-rate and body mass.

### (f) Life-history trade-offs

The well-known trade-off relationship between mass and development time was not consistently observed across treatments. Instead, the slope of the relationship ranged from positive, as expected under a trade-off scenario, to weakly negative or nearly zero, depending on the combination of temperature and photoperiod. For example, a clear trade-off was detected under a constant high temperature, as used in the constant warmer temp + constant photop treatment. Here, the stable and permissive rearing conditions may have allowed larvae to allocate resources efficiently [62], producing the expected trade-off between mass and development time in the absence of seasonal photoperiodic cues. By contrast, under seasonal temperature and photoperiod regimes, as in the seasonal ambient temp + seasonal photop and seasonal warmer temp + seasonal photop treatments, the relationship between mass and development time was weakly negative. This suggests that individuals did not accelerate development at the expense of mass. Instead, other physiological or energy constraints may have decoupled mass and development time. Consequently, the expected temperature–size relationship was not evident in these treatments. Interestingly, we found a positive relationship (trade-off) in the seasonal ambient temp + constant photop treatment, for which we currently have no clear explanation. In any case, these results demonstrate that the trade-off relationship depends strongly on the specific combination of temperature and photoperiod used.

To conclude, our study demonstrated the importance of considering seasonal temperatures and photoperiods when assessing the effects of warming on our study species. While our proof-of-evidence study relied on a single species, it provides a foundation for broader comparative research on ectotherms. Given that the responses observed in our study species closely match both theoretical predictions and empirical observations of ectotherm responses to warming and photoperiod, the response patterns identified here are likely to be broadly generalizable.

## Supplementary Material



## Data Availability

All data are available at Dryad [72] . Supplementary material is available online [73].

## References

[1] Angilletta Jr MJ . 2009 Thermal adaptation: a theoretical and empirical synthesis. New York, NY: Oxford University Press.

[2] Andersson M . 1994 Sexual selection. Princeton, NJ: Princeton University Press.

[3] Sentis A , Bazin S, Boukal DS, Stoks R. 2024 Ecological consequences of body size reduction under warming. Proc. R. Soc. B 291, 20241250. (doi:10.1098/rspb.2024.1250) PMC11337126 39166384

[4] Denny M . 2017 The fallacy of the average: on the ubiquity, utility and continuing novelty of Jensen's inequality. J. Exp. Biol. 220, 139–146. (doi:10.1242/jeb.140368) 28100801

[5] Vasseur DA , DeLong JP, Gilbert B, Greig HS, Harley CDG, McCann KS, Savage V, Tunney TD, O'Connor MI. 2014 Increased temperature variation poses a greater risk to species than climate warming. Proc. R. Soc. B 281, 20132612. (doi:10.1098/rspb.2013.2612) PMC3924069 24478296

[6] Williams CT , Klaassen M, Barnes BM, Buck CL, Arnold W, Giroud S, Vetter SG, Ruf T. 2017 Seasonal reproductive tactics: annual timing and the capital-to-income breeder continuum. Phil. Trans. R. Soc. B 372, 20160250. (doi:10.1098/rstb.2016.0250) PMC5647277 28993494

[7] Bradshaw WE , Holzapfel CM. 2007 Evolution of animal photoperiodism. Annu. Rev. Ecol. Evol. Syst. 38, 1–25. (doi:10.1146/annurev.ecolsys.37.091305.110115)

[8] Tauber MJ , Tauber CA, Masaki S. 1986 Seasonal adaptations of insects. New York, NY: Oxford University Press.

[9] Sniegula S , Stoks R, Golab MJ. 2024 Insect responses to seasonal time constraints under global change are facilitated by warming and counteracted by invasive alien predators. Sci. Rep. 14, 24565. (doi:10.1038/s41598-024-76057-x) PMC11490650 39427019

[10] Verspagen N , Ikonen S, Maes D, Stefanescu C, DiLeo MF, Saastamoinen M. 2023 Thermal plasticity in development and diapause strategy in a temperate butterfly across a latitudinal gradient. Funct. Ecol. 37, 3111–3123. (doi:10.1111/1365-2435.14446)

[11] Li X , Ye Z, Chen J, Zhu J, Han D, Wang J, Li L, Fu Y, Zhang F. 2023 Influence of temperature, photoperiod, and supplementary nutrition on the development and reproduction of *Scutellista caerulea* Fonscolombe (Hymenoptera: Pteromalidae). Insects 14, 82. (doi:10.3390/insects14010082) PMC9861902 36662009

[12] Koštál V . 2006 Eco-physiological phases of insect diapause. J. Insect Physiol. 52, 113–127. (doi:10.1016/j.jinsphys.2005.09.008) 16332347

[13] Lankinen P , Kastally C, Hoikkala A. 2023 Clinal variation in the temperature and photoperiodic control of reproductive diapause in *Drosophila montana* females. J. Insect Physiol. 150, 104556. (doi:10.1016/j.jinsphys.2023.104556) 37598869

[14] Śniegula S , Gołąb MJ, Johansson F. 2016 Time constraint effects on phenology and life history synchrony in a damselfly along a latitudinal gradient. Oikos 125, 414–423. (doi:10.1111/oik.02265)

[15] Shama LNS , Campero‐Paz M, Wegner KM, De Block M, Stoks R. 2011 Latitudinal and voltinism compensation shape thermal reaction norms for growth rate. Mol. Ecol. 20, 2929–2941. (doi:10.1111/j.1365-294X.2011.05156.x) 21689189

[16] Beuvard C , Imsland AKD, Thorarensen H. 2022 The effect of temperature on growth performance and aerobic metabolic scope in Arctic charr, *Salvelinus alpinus* (L.). J. Therm. Biol. 104, 103117. (doi:10.1016/j.jtherbio.2021.103117) 35180951

[17] Leimar O . 1996 Life history plasticity: influence of photoperiod on growth and development in the common blue butterfly. Oikos 76, 228–234. (doi:10.2307/3546194)

[18] Lindgren B , Laurila A. 2005 Proximate causes of adaptive growth rates: growth efficiency variation among latitudinal populations of *Rana temporaria*. J. Evol. Biol. 18, 820–828. (doi:10.1111/j.1420-9101.2004.00875.x) 16033553

[19] Masaki S , Walker TJ. 1987Cricket life cycles. In *Evolutionary Biology* (eds Hecht MK, Wallace B, Prance GT), pp. 349–423. Boston, MA: Springer. (doi:10.1007/978-1-4615-6986-2_11)

[20] Śniegula S , Drobniak SM, Gołąb MJ, Johansson F. 2014 Photoperiod and variation in life history traits in core and peripheral populations in the damselfly *Lestes sponsa*. Ecol. Entomol. 39, 137–148. (doi:10.1111/een.12076)

[21] Nagano K , Hiraiwa MK, Ishiwaka N, Seko Y, Hashimoto K, Uchida T, Sánchez-Bayo F, Hayasaka D. 2023 Global warming intensifies the interference competition by a poleward-expanding invader on a native dragonfly species. R. Soc. Open Sci. 10, 230449. (doi:10.1098/rsos.230449) PMC10663793 38026017

[22] Atkinson D . 1994 Temperature and organism size—a biological law for ectotherms? Adv. Ecol. Res. 25, 1–58. (doi:10.1016/S0065-2504(08)60212-3)

[23] Rowe L , Ludwig D. 1991 Size and timing of metamorphosis in complex life histories: time constraints and variation. Ecology 72, 413–427. (doi:10.2307/2937184)

[24] Roff DA . 1992 The evolution of life histories: theory and analysis. New York, NY: Chapman & Hall, Inc.

[25] Frances DN , Moon JY, McCauley SJ. 2017 Effects of environmental warming during early life history on libellulid odonates. Can. J. Zool. 95, 373–382. (doi:10.1139/cjz-2016-0233)

[26] Kukita S , Gouda M, Ikeda S, Ishibashi S, Furuya T, Nakamura K. 2015 Effects of photoperiod and temperature on growth and development in clouded salamander (*Hynobius nebulosus*) larvae. Zool. Sci. 32, 266–271. (doi:10.2108/zs140220) 26003982

[27] Palomar G , Wos G, Stoks R, Sniegula S. 2023 Latitude-specific urbanization effects on life history traits in the damselfly *Ischnura elegans*. Evol. Appl. 16, 1503–1515. (doi:10.1111/eva.13583) PMC10445092 37622092

[28] Scharf I , Bauerfeind SS, Blanckenhorn WU, Schäfer MA. 2010 Effects of maternal and offspring environmental conditions on growth, development and diapause in latitudinal yellow dung fly populations. Clim. Res. 43, 115–125. (doi:10.3354/cr00907)

[29] Siemers I , von Schmalensee L, Ziesemer S, Gotthard K, Lehmann P. 2024 Predictable local adaptation in butterfly photoperiodism but not thermal performance along a latitudinal cline. Ecosphere 15, e4842. (doi:10.1002/ecs2.4842)

[30] Dańko MJ , Dańko A, Golab MJ, Stoks R, Sniegula S. 2017 Latitudinal and age-specific patterns of larval mortality in the damselfly *Lestes sponsa*: senescence before maturity? Exp. Gerontol. 95, 107–115. (doi:10.1016/j.exger.2017.05.008) 28502774

[31] Song YH . 2016 The effect of fluctuations in photoperiod and ambient temperature on the timing of flowering: time to move on natural environmental conditions. Mol. Cells 39, 715–721. (doi:10.14348/molcells.2016.0237) PMC5104878 27788575

[32] Walker II WH, Meléndez‐Fernández OH, Nelson RJ, Reiter RJ. 2019 Global climate change and invariable photoperiods: a mismatch that jeopardizes animal fitness. Ecol. Evol. 9, 10044–10054. (doi:10.1002/ece3.5537) PMC6745832 31534712

[33] Amer NR , Golab MJ, Stoks R, Wos G, Sniegula S. 2025 Time constraints modulate the effects of predator cues and a metal across life stages in a damselfly. Evol. Appl. 18, e70169. (doi:10.1111/eva.70169) PMC12577438 41181520

[34] Norling U . 2021 Growth, winter preparations and timing of emergence in temperate zone Odonata: control by a succession of larval response patterns. Int. J. Odonatol. 24, 1–36. (doi:10.23797/2159-6719_24_1)

[35] Neptune TC , Benard MF. 2024 Longer days, larger grays: carryover effects of photoperiod and temperature in gray treefrogs, *Hyla versicolor*. Proc. R. Soc. B 291, 20241336. (doi:10.1098/rspb.2024.1336) PMC11335022 38981527

[36] Wos G , Amer NR, Antoł A, Stoks R, Sniegula S. 2025 Warming and latitude shape the non-consumptive effects of native and invasive alien crayfish predators on damselfly prey. NeoBiota 98, 223–245. (doi:10.3897/neobiota.98.141133)

[37] Sniegula S , Golab MJ, Johansson F. 2019 Size-mediated priority and temperature effects on intra-cohort competition and cannibalism in a damselfly. J. Anim. Ecol. 88, 637–648. (doi:10.1111/1365-2656.12947) 30659605

[38] L Hyeun-Ji , Johansson F. 2016 Compensating for a bad start: compensatory growth across life stages in an organism with a complex life cycle. Can. J. Zool. 94, 41–47. (doi:10.1139/cjz-2015-0157)

[39] Sokolovska N , Rowe L, Johansson F. 2000 Fitness and body size in mature odonates. Ecol. Entomol. 25, 239–248. (doi:10.1046/j.1365-2311.2000.00251.x)

[40] Waller JT , Svensson EI. 2017 Body size evolution in an old insect order: no evidence for Cope's Rule in spite of fitness benefits of large size. Evolution 71, 2178–2193. (doi:10.1111/evo.13302) 28685868

[41] Banks MJ , Thompson DJ. 1987 Regulation of damselfly populations: the effects of larval density on survival, development rate and size in the field. Freshw. Biol. 17, 357–365. (doi:10.1111/j.1365-2427.1987.tb01055.x)

[42] Boudot J-P , Kalkman VJ. 2015 Atlas of the European dragonflies and damselflies. Zeist, The Netherlands: KNNV Publishing.

[43] Corbet PS . 1956 The life-histories of *Lestes sponsa* (Hansemann) and *Sympetrum striolatum* (Charpentier)(Odonata). Tijdschr. Entomol. 99, 217–229.

[44] Corbet PS . 1956 The influence of temperature on diapause development in the dragonfly *Lestes sponsa* (Hansemann) (Odonata: Lestidae). Proc. R. Entomol. Soc. A 31, 45–48. (doi:10.1111/j.1365-3032.1956.tb00205.x)

[45] Norling U , Sniegula S. 2025 Maintenance and termination of the embryonic diapause in the univoltine damselfly *Lestes sponsa* Hansemann. Res. Sq. (doi:10.21203/rs.3.rs-7939953/v1)

[46] Sniegula S , Golab MJ, Drobniak SM, Johansson F. 2016 Seasonal time constraints reduce genetic variation in life-history traits along a latitudinal gradient. J. Anim. Ecol. 85, 187–198. (doi:10.1111/1365-2656.12442) 26333659

[47] De Block M , Stoks R. 2005 Fitness effects from egg to reproduction: bridging the life history transition. Ecology 86, 185–197. (doi:10.1890/04-0116)

[48] Krishnaraj R , Pritchard G. 1995 The influence of larval size, temperature, and components of the functional response to prey density on growth rates of the dragonflies *Lestes disjunctus* and *Coenagrion resolutum* (Insecta: Odonata). Can. J. Zool. 73, 1672–1680. (doi:10.1139/z95-199)

[49] Norling U . 2018 Constant and shifting photoperiods as seasonal cues during larval development of the univoltine damselfly *Lestes sponsa* (Odonata: Lestidae). Int. J. Odonatol. 21, 129–150. (doi:10.1080/13887890.2018.1462263)

[50] Nilsson-Örtman V , Stoks R, Johansson F. 2014 Competitive interactions modify the temperature dependence of damselfly growth rates. Ecology 95, 1394–1406. (doi:10.1890/13-0875.1) 25000770

[51] Nylin S , Wickman P-O, Wiklund C. 1989 Seasonal plasticity in growth and development of the speckled wood butterfly, *Pararge aegeria* (Satyrinae). Biol. J. Linn. Soc. 38, 155–171. (doi:10.1111/j.1095-8312.1989.tb01571.x)

[52] Stoks R , De Block M. 2011 Rapid growth reduces cold resistance: evidence from latitudinal variation in growth rate, cold resistance and stress proteins. PLoS ONE 6, e16935. (doi:10.1371/journal.pone.0016935) PMC3044720 21390210

[53] Tian R , Hou Z, Li S, Chai H. 2024 Effects of photoperiod and temperature on the developmental duration and diapause in *Diolycoris baccarum* (Heteroptera: Pentatomidae) from Hohhot, Inner Mongolia, China. J. Insect Sci. 24, 2. (doi:10.1093/jisesa/ieae025) PMC10914362 38442351

[54] Yuan X , Wei S, Li D, Zhang J. 2023 Lighting in dark periods reduced the fecundity of *Spodoptera frugiperda* and limited its population growth. Agronomy 13, 971. (doi:10.3390/agronomy13040971)

[55] Mikolajewski DJ , Johansson F, Brodin T. 2004 Condition-dependent behaviour among damselfly populations. Can. J. Zool. 82, 653–659. (doi:10.1139/Z04-036)

[56] Sniegula S , Raczyński M, Golab MJ, Johansson F. 2020 Effects of predator cues carry over from egg and larval stage to adult life-history traits in a damselfly. Freshw. Sci. 39, 804–811. (doi:10.1086/711374)

[57] Forster J , Hirst AG, Atkinson D. 2012 Warming-induced reductions in body size are greater in aquatic than terrestrial species. Proc. Natl Acad. Sci. USA 109, 19310–19314. (doi:10.1073/pnas.1210460109) PMC3511100 23129645

[58] Siepielski AM , Hasik AZ, Ping T, Serrano M, Strayhorn K, Tye SP. 2020 Predators weaken prey intraspecific competition through phenotypic selection. Ecol. Lett. 23, 951–961. (doi:10.1111/ele.13491) 32227439

[59] R Core Team. 2025 R: a language and environment for statistical computing. Vienna, Austria: R Foundation for Statistical Computing. https://www.R-project.org/.

[60] Serediuk H , Jackson J, Evers SM, Paniw M. 2024 Comparative life-history responses of lacewings to changes in temperature. Ecol. Evol. 14, e70000. (doi:10.1002/ece3.70000) PMC11257770 39026964

[61] Flenner I , Richter O, Suhling F. 2010 Rising temperature and development in dragonfly populations at different latitudes. Freshw. Biol. 55, 397–410. (doi:10.1111/j.1365-2427.2009.02289.x)

[62] Stoks R , Swillen I, De Block M. 2012 Behaviour and physiology shape the growth accelerations associated with predation risk, high temperatures and southern latitudes in *Ischnura* damselfly larvae. J. Anim. Ecol. 81, 1034–1040. (doi:10.1111/j.1365-2656.2012.01987.x) 22524392

[63] Walter JA , Thompson LM, Powers SD, Parry D, Agosta SJ, Grayson KL. 2022 Growth and development of an invasive forest insect under current and future projected temperature regimes. Ecol. Evol. 12, e9017. (doi:10.1002/ece3.9017) PMC9204848 35784073

[64] McCauley SJ , Hammond JI, Frances DN, Mabry KE. 2014 Effects of experimental warming on survival, phenology, and morphology of an aquatic insect (Odonata). Ecol. Entomol. 40, 211–220. (doi:10.1111/een.12175) PMC4443926 26028806

[65] Suhling I , Suhling F. 2013 Thermal adaptation affects interactions between a range-expanding and a native odonate species. Freshw. Biol. 58, 705–714. (doi:10.1111/fwb.12074)

[66] Suhling F , Suhling I, Richter O. 2015 Temperature response of growth of larval dragonflies – an overview. Intern. J. Odonatol. 18, 15–30. (doi:10.1080/13887890.2015.1009392)

[67] De Block M , Stoks R. 2003 Adaptive sex-specific life history plasticity to temperature and photoperiod in a damselfly. J. Evol. Biol. 16, 986–995. (doi:10.1046/j.1420-9101.2003.00581.x) 14635914

[68] Johansson F , Rowe L. 1999 Life history and behavioral responses to time constraints in a damselfly. Ecology 80, 1242–1252. (doi:10.1890/0012-9658(1999)080[1242:LHABRT]2.0.CO;2)

[69] Sniegula S , Golab MJ, Johansson F. 2016 A large-scale latitudinal pattern of life-history traits in a strictly univoltine damselfly. Ecol. Entomol. 41, 459–472. (doi:10.1111/een.12314)

[70] Teder T , Taits K, Kaasik A, Tammaru T. 2022 Limited sex differences in plastic responses suggest evolutionary conservatism of thermal reaction norms: a meta-analysis in insects. Evol. Lett. 6, 394–411. (doi:10.1002/evl3.299) PMC9783480 36579171

[71] Arnqvist G , Rowe L. 2005 Sexual conflict. Princeton, NJ: Princeton University Press.

[72] Johansson F , Stoks R, Wos G, Sniegula S. 2026 Data from: Constant versus seasonal photoperiod and temperature yield divergent life-history responses to climate warming. Dryad Digital Repository. (doi:10.5061/dryad.rv15dv4n6) PMC13725532 42947425

[73] Johansson F , Stoks R, Wos G, Sniegula S. 2026 Supplementary material from: Constant versus seasonal photoperiod and temperature yield divergent life-history responses to climate warming. Figshare. (doi:10.6084/m9.figshare.c.8672764) PMC13725532 42947425

