# Supplementary material for: Constant versus seasonal photoperiod and temperature yield divergent life-history responses to climate warming
Source: Proc Biol Sci. 2026 Oct 28;293(2081):20261212. doi: 10.1098/rspb.2026.1212 (PMC13725532; doi:10.1098/rspb.2026.1212)
Supplement: Supplementary file 1 [file rspb.2026.1212.zip › rspb.2026.1212/rspb20261212_si_001.docx]

Authors: Frank Johansson, Robby Stoks, Guillaume Wos and Szymon Sniegula

Title: Constant versus seasonal photoperiod and temperature yield divergent life-history responses to climate warming

Journal: Proceedings of the Royal Society B

DOI: https://doi.org/10.1098/rspb.2026.1212

Supplementary Figure

Figure S2. Synchrony of larval development time until entrance into the F-0 in *L. sponsa*. The synchrony was measured as a coefficient of variation (CV); lower values indicated higher synchrony. Abbreviations: CAT+CP—constant ambient temperature and constant photoperiod; CWT+CP—constant warmer temperature and constant photoperiod; SAT+SP—seasonal ambient temperature and seasonal photoperiod; SWT+SP—seasonal warmer temperature and seasonal photoperiod; CAT+SP—constant ambient temperature and seasonal photoperiod; SAT+CP—seasonal ambient temperature and constant photoperiod.
